# Kalman/Map Filtering-Aided Fast Normalized Cross Correlation-Based Wi-Fi Fingerprinting Location Sensing

**DOI:** 10.3390/s131115513

**Published:** 2013-11-13

**Authors:** Yongliang Sun, Yubin Xu, Cheng Li, Lin Ma

**Affiliations:** 1 School of Electronics and Information Engineering, Harbin Institute of Technology, Harbin 150001, China; E-Mails: syl_peter@163.com (Y.S.); malin@hit.edu.cn (L.M.); 2 Faculty of Engineering and Applied Science, Memorial University, St. John's, NL A1B 3X5, Canada; E-Mail: licheng@mun.ca

**Keywords:** indoor location sensing, Wi-Fi fingerprinting, fast normalized cross correlation, map matching, Kalman/map filtering

## Abstract

A Kalman/map filtering (KMF)-aided fast normalized cross correlation (FNCC)-based Wi-Fi fingerprinting location sensing system is proposed in this paper. Compared with conventional neighbor selection algorithms that calculate localization results with received signal strength (RSS) mean samples, the proposed FNCC algorithm makes use of all the on-line RSS samples and reference point RSS variations to achieve higher fingerprinting accuracy. The FNCC computes efficiently while maintaining the same accuracy as the basic normalized cross correlation. Additionally, a KMF is also proposed to process fingerprinting localization results. It employs a new map matching algorithm to nonlinearize the linear location prediction process of Kalman filtering (KF) that takes advantage of spatial proximities of consecutive localization results. With a calibration model integrated into an indoor map, the map matching algorithm corrects unreasonable prediction locations of the KF according to the building interior structure. Thus, more accurate prediction locations are obtained. Using these locations, the KMF considerably improves fingerprinting algorithm performance. Experimental results demonstrate that the FNCC algorithm with reduced computational complexity outperforms other neighbor selection algorithms and the KMF effectively improves location sensing accuracy by using indoor map information and spatial proximities of consecutive localization results.

## 1. Introduction

As the development and integration of mobile computing and sensing technologies, ubiquitous computing integrates the physical world and information space and offers widely available computing and information services [1]. In ubiquitous computing, location-sensing computing is becoming increasingly important due to most services are location-based services (LBSs) [2], such as vehicular and asset management, emergency rescue, pedestrian navigation, and proximity marketing. These services are relative to mobile terminal locations. Outdoor users can obtain accurate location information through satellite localization systems [3], but satellite localization system performance is limited in indoor environments owing to signal attenuation and complex radio propagation. Cellular localization also cannot achieve the needed accuracy for indoor LBSs [4]. In this light, numerous indoor location sensing systems have been developed by employing different technologies, such as Bluetooth [5], infrared [6], radio-frequency identification [7], ultra wideband [8], ultrasound [9], and Wi-Fi received signal strength (RSS) [10,11]. Currently, Wi-Fi RSS fingerprinting location sensing is specially preferred and extensively researched because RSS samples are easily sensed and collected by commonly used Wi-Fi mobile terminals from pervasively deployed access points (APs) without additional hardware being required [12].

The RSS fingerprinting method exploits a nonlinear mapping relationship between physical locations and RSS samples from multiple APs. In the off-line phase of the fingerprinting method, specific locations called reference points (RPs) are labeled and RSS samples collected at these RPs are recorded in a database called radiomap. In the on-line phase, on-line RSS data are collected by a Wi-Fi mobile terminal and matched with the RSS data in the radiomap to estimate localization results [13]. So far, many algorithms have been used for RSS fingerprinting. These fingerprinting algorithms are mainly classified as: machine learning and neighbor selection algorithms [14]. The machine learning algorithms, such as artificial neural network (ANN) [15], adaptive neural-fuzzy inference system (ANFIS) [16] and support vector machine (SVM) [17], need an off-line training to model the nonlinear mapping relationship between RSS data and RP location coordinates. Localization coordinates are estimated by the trained nonlinear mapping function with on-line RSS mean samples. Nearest neighbor, K nearest neighbors (KNN) and weighted KNN (WKNN) algorithms are conventional neighbor selection algorithms [11,14]. They select RPs according to RSS distances between the on-line RSS mean sample and RSS mean samples of all the RPs and then estimate localization coordinates with the selected RP coordinates.

However, using the existing fingerprinting algorithms, multiple on-line RSS samples collected at one location are averaged to compute an RSS mean sample, so useful on-line RSS information is lost in the RSS mean computation. These fingerprinting algorithms fail to make full use of all the on-line RSS information [11, 15–17]. Additionally, some other available information can also be employed for location-sensing computing, like RSS variations of RPs, indoor map information and spatial proximities of consecutive localization results.

Thus, to take advantage of all this information for accuracy improvement, the contributions of this paper are summarized as follows:

First, a fast normalized cross correlation (FNCC) fingerprinting algorithm is proposed to calculate localization coordinates with all the collected on-line RSS samples. It also regards RSS variations of RPs as weights for correlation coefficient computations to precisely select RPs. Compared with the basic normalized cross correlation (NCC) algorithm, which is computationally expensive, the proposed FNCC algorithm greatly reduces computational complexity while maintaining the same accuracy as the NCC, which is higher than those of conventional neighbor selection algorithms.

Second, indoor map information is used by a proposed map matching algorithm. A calibration model that represents pedestrian walkways is integrated into an indoor map. Then a map image matrix is created for the map matching algorithm. Based on the building interior structure, unreasonable location coordinates are corrected by the map matching algorithm to the calibration model and therefore more accurate location coordinates are obtained.

Third, based on the map matching algorithm, a Kalman/map filtering (KMF) is proposed to process fingerprinting results using indoor map information and spatial proximities of consecutive localization results. Through nonlinearizing the linear prediction process of Kalman filtering (KF) by the map matching algorithm, more accurate prediction locations are obtained for the KMF. This greatly improves the KMF performance of increasing location sensing accuracy.

The remainder of this paper is structured as follows: in Section 2, related work is discussed. The proposed FNCC fingerprinting algorithm, map matching algorithm and KMF are described in detail in Section 3. Section 4 gives the experimental setup, results and analyses. Finally, the paper is concluded in Section 5.

## 2. Related Work

To the best of our knowledge, NCC has not been applied as an RSS fingerprinting algorithm for location-sensing computing. However, Xiao *et al.* used correlation coefficients to quantify similarities between observed and stored channel state information to measure the distances between a mobile terminal and RPs [18]. Liu *et al.* computed spatial correlation between an RP and scanning points (SPs) in the same micro cell [19]. The measured RSS samples at the SPs were used to estimate the RSS data of the RP for a micro-cell radiomap construction.

In the area of image similarity measurements, NCC has been used extensively [20,21]. Because the basic NCC algorithm is time-consuming and is not suitable for time-critical applications, several fast NCC algorithms have been developed to improve computational efficiency. Lewis proposed a fast NCC algorithm based on a sum table approach [22]. But the sum table approach could only efficiently calculate the NCC denominator. It could not be directly applied to calculate the numerator. Yoo *et al.* proposed a fast NCC algorithm without using multiplication operations [23]. Under an assumption made for the fast algorithm, the simplified method saved computational complexity at the expense of degrading algorithm performance. When their proposed simplified method was applied to the NCC-based fingerprinting algorithm in this paper, it could not precisely measure similarities between on-line RSS data and RSS data in the radiomap. Wei *et al.* employed another improved NCC for image template matching [24]. The basic NCC equation was rewritten with the image mean substitution operations. The computational efficiency of the rewritten NCC was limitedly increased. Therefore, based on the RSS fingerprinting theory, the proposed FNCC fingerprinting algorithm is able to achieve the same high accuracy as the basic NCC algorithm with greatly reduced computational complexity.

Regarding the filtering algorithms for processing Wi-Fi fingerprinting results, particle filtering (PF) and KF are commonly used. As a linear filtering algorithm, KF processed the localization results of the ANN, nearest neighbor and propagation model [25–27] by using spatial proximities of consecutive localization results. But the KF linear state prediction is usually not accurate, which limits its performance. With measurement data from sensors, like electronic compasses, accelerometers or odometers, nonlinear PF improved localization results with the fused sensor data [28,29]. Moreover, indoor map information was also combined with PF-based location sensing systems to further improve system accuracy. Leppakoski *et al.* employed a PF to fuse Wi-Fi fingerprinting location estimates with pedestrian dead reckoning (PDR) sensor data and map information for pedestrian indoor navigation [30]. Their experimental results indicated that even the poor quality Wi-Fi fingerprinting location estimates still included useful information and fusing them with the sensor data and map information improved their system performance. Beauregard *et al.* developed a backtracking PF combined with wall information and PDR sensor data to correct the heading direction of a user [31]. With the wall information, particles tried to go across walls were abandoned. The previous state estimate was recalculated without these invalid particle trajectories, so a better estimate was produced. Ascher *et al.* proposed a map matching algorithm based on PF for 3D map matching [32]. Accurate height estimation from inertial measurement unit and barometer measurements was used for particle constraints to match the estimated trajectory to a multi-floor map.

However, PF-based algorithms are usually time-consuming, especially when the number of particles is considerable. Compared with the PF-based algorithms, the KF computes much faster. Because it works iteratively and just requires a prediction state and measurement data. Thus, through combining a presented map matching algorithm and the KF, a KMF is proposed in this paper. The KF linear prediction process is nonlinearized by the map matching algorithm. With the advantages of both the map matching algorithm and KF, the proposed KMF effectively improves location sensing accuracy with only Wi-Fi fingerprinting results as measurement data. Regarding the limited hardware resources and power of Wi-Fi mobile terminals, the KMF is suitable to be embedded into the Wi-Fi mobile terminals for practical applications.

## 3. Kalman/Map Filtering-Aided Fast Normalized Cross Correlation-Based Wi-Fi Fingerprinting Location Sensing

In Section 3.1, the proposed FNCC fingerprinting algorithm is described in detail. The FNCC not only makes full use of off-line and on-line RSS information, but also greatly reduces computational complexity, especially for calculating the NCC denominator, while maintaining high fingerprinting accuracy. A map matching algorithm is presented in Section 3.2. An indoor map is processed and converted into an image matrix with a calibration model for coordinate corrections. With the image matrix, the map matching algorithm can be used as an independent algorithm to correct unreasonable location coordinates. With the integration of the map matching algorithm into the KF, the proposed KMF algorithm is detailed in Section 3.3. The KMF effectively improves location sensing accuracy with indoor map information and spatial proximities of consecutive localization results. For convenience, key symbols used in this paper are listed in Table 1.

### 3.1. Fast Normalized Cross Correlation-Based Fingerprinting Algorithm

NCC has been widely used for many image processing applications because of its abilities to cope with noisy images. In this paper, it is applied to measure the correlation degree between collected on-line RSS data and RSS data of an RP using a correlation coefficient, which ranges from −1 to 1. The correlation coefficient is equal to −1 in the case of a perfect negative correlation, 0 no correlation and 1 a perfect positive correlation [23]. The closer the correlation coefficient approaches to −1 or 1, the stronger the correlation degree between the two RSS data sets. Compared with existing fingerprinting algorithms that calculate localization results with RSS mean samples, the NCC-based fingerprinting algorithm takes advantage of all the available on-line RSS samples to avoid losing useful RSS information in the process of computing an RSS mean sample. In addition, the algorithm also integrates RSS variations of RPs into correlation computations. Thus, the RPs that have more similar RSS characteristics with the collected on-line RSS data can be precisely selected for location-sensing computing. However, the basic NCC algorithm is computationally expensive, so an FNCC fingerprinting algorithm is presented to reduce computational complexity and speed up correlation computations. The correlation coefficient *r_l_* of the basic NCC between the on-line RSS matrix **rss** and the RSS matrix ***RSS****_l_* of *l* th RP can be calculated by:

(1) $$r_{l}=\frac{\overset{N}{\underset{j=1}{\text{∑}}}\overset{M}{\underset{i=1}{\text{∑}}}\left({\text{rss}}_{i,j}-\mu_{\mathrm{rss}}\right)\left({\text{RSS}}_{i,j,l}-\mu_{l}\right)}{\sqrt{\overset{N}{\underset{j=1}{\text{∑}}}\overset{M}{\underset{i=1}{\text{∑}}}{\left({\text{rss}}_{i,j}-\mu_{\mathrm{rss}}\right)}^{2}}\sqrt{\overset{N}{\underset{j=1}{\text{∑}}}\overset{M}{\underset{i=1}{\text{∑}}}{\left({\text{RSS}}_{i,j,l}-\mu_{l}\right)}^{2}}},l=1,2,\cdots ,L$$

where parameters *μ*_rss_ and *μ_l_* are the means of the RSS matrices **rss** and ***RSS****_l_*, respectively. They are computed by Equation (2) as follows:

(2) $$\left\{ \begin{array}{l} \mu_{\mathrm{rss}}=\frac{1}{N\times M}\overset{N}{\underset{j=1}{\text{∑}}}\overset{M}{\underset{i=1}{\text{∑}}}{\text{rss}}_{i,j}\\ \mu_{l}=\frac{1}{N\times M}\overset{N}{\underset{j=1}{\text{∑}}}\overset{M}{\underset{i=1}{\text{∑}}}{\text{RSS}}_{i,j,l} \end{array}\right.,l=1,2,\cdots ,L$$

After all these *L* correlation coefficients are obtained, they are sorted in descending order. The RPs that correspond to the first *K* maximum correlation coefficients are selected to compute localization coordinates through averaging the selected RP location coordinates, which is denoted by:

(3) $$\left\{ \begin{array}{l} \left(\bar{x},\bar{y}\right)=\frac{1}{K}\overset{K}{\underset{i=1}{\text{∑}}}\left(x_{i},y_{i}\right)=\frac{1}{K}\overset{K}{\underset{i=1}{\text{∑}}}{\text{loc}}_{i}\\ {\text{loc}}_{i}=\left(x_{i},y_{i}\right),r_{i}\in \left\{\mathrm{MAX}\_K\left(r_{1},\cdots ,r_{L}\right)\right\} \end{array}\right.,i=1,2,\cdots ,K$$

where ***loc****_i_* is the coordinate vector of the selected RP *i*, {*MAX_K*(*r_1_*,…, *r_L_*)} is the set of the first *K* maximum correlation coefficients and (*x̄*,*ȳ*) are the localization coordinates.

For computing one localization result, the *L* correlation coefficients are calculated with the same on-line RSS matrix, so the term $\sqrt{\overset{N}{\underset{j=1}{\text{∑}}}\overset{M}{\underset{i=1}{\text{∑}}}{\left({\text{rss}}_{i,j}-\mu_{\mathrm{rss}}\right)}^{2}}$ in Equation (1) that is only relative to the on-line RSS matrix can be regarded as a constant. Without changing the order of the *L* correlation coefficients, Equation (1) can be simplified as:

(4) $$r_{l}=\frac{\overset{N}{\underset{j=1}{\text{∑}}}\overset{M}{\underset{i=1}{\text{∑}}}\left({\text{rss}}_{i,j}-\mu_{\text{rss}})({\text{RSS}}_{i,j,l}-\mu_{l}\right)}{\sqrt{\overset{N}{\underset{j=1}{\text{∑}}}{\overset{M}{\underset{i=1}{\text{∑}}}\left({\text{RSS}}_{i,j,l}-\mu_{l}\right)}^{2}}},l=1,2,\cdots ,L$$

As we know, it is much faster for a computer to execute a multiplication operation than to execute a square root operation, so Equation (4) is squared to substitute the square root operation in denominator with a multiplication operation in numerator. The squared correlation coefficient $r_{l}^{2}$ is computed by:

(5) $$r_{l}^{2}=\frac{{\left(\overset{N}{\underset{j=1}{\text{∑}}}\overset{M}{\underset{i=1}{\text{∑}}}\left({\text{rss}}_{i,j}-\mu_{\mathrm{rss}}\right)\left({\text{RSS}}_{i,j,l}-\mu_{l}\right)\right)}^{2}}{\overset{N}{\underset{j=1}{\text{∑}}}\overset{M}{\underset{i=1}{\text{∑}}}{\left({\text{RSS}}_{i,j,l}-\mu_{l}\right)}^{2}},l=1,2,\cdots ,L$$

Compared with the basic NCC computation denoted by Equation (1), Equation (5) saves *N* × *M* − *1* addition/subtraction operations, *N* × *M* multiplication/division operations and 2 square root operations for computing one correlation coefficient. When the means *μ*_rss_ and *μ_l_* in Equation (5) are substituted with Equation (2), the squared correlation coefficient $r_{l}^{2}$ is also calculated by:

(6) $$r_{l}^{2}=\frac{{\left(\overset{N}{\underset{j=1}{\text{∑}}}\overset{M}{\underset{i=1}{\text{∑}}}{\text{rss}}_{i,j}{\text{RSS}}_{i,j,l}-\frac{1}{N\times M}\overset{N}{\underset{j=1}{\text{∑}}}\overset{M}{\underset{i=1}{\text{∑}}}{\text{rss}}_{i,j}\overset{N}{\underset{j=1}{\text{∑}}}\overset{M}{\underset{i=1}{\text{∑}}}{\text{RSS}}_{i,j,l}\right)}^{2}}{\overset{N}{\underset{j=1}{\text{∑}}}\overset{M}{\underset{i=1}{\text{∑}}}{{\text{RSS}}_{i,j,l}}^{2}-\frac{1}{N\times M}{\left(\overset{N}{\underset{j=1}{\text{∑}}}\overset{M}{\underset{i=1}{\text{∑}}}{\text{RSS}}_{i,j,l}\right)}^{2}},l=1,2,\cdots ,L$$

For calculating one squared correlation coefficient $r_{l}^{2}$, Equation (6) executes *2* × *N* × *M* − *2* less addition/subtraction operations and 2 more multiplication/division operations than Equation (5). Usually, Equation (6) computes faster than Equation (5), especially under conditions when the numbers of deployed APs *M* and collected on-line RSS samples *N* are considerable.

### 3.2. Indoor Map Matching for Coordinate Corrections

To improve location sensing performance, an indoor map integrated with a calibration model is processed for the proposed map matching algorithm. The indoor map is derived from an indoor building plan of computer aided design (CAD) and the calibration model is comprised of straight lines that represent pedestrian walkways. According to the building interior structure, unreasonable localization results are corrected to the calibration model for accuracy improvement.

First, the original indoor map and the indoor map integrated with the calibration model are saved as two JPEG gray-scale images **G**_model_ and **G**_no_model_, respectively. Using Equation (7), the two images are converted into two binary images **I**_model_ and **I**_no_model_, respectively. The size of the binary image matrices is proportionally adapted according to the size of the building plan.

(7) $$\text{I}\left(p^{x},p^{y}\right)=\left\{ \begin{array}{l} 1,\text{G}\left(p^{x},p^{y}\right)\geq T_{\text{threshold}}\\ 0,\text{G}\left(p^{x},p^{y}\right)<T_{\text{threshold}} \end{array}\right.$$

where ***T***_threshold_ is the threshold.

In the two binary images **I**_model_ and **I**_no_model_, the pixel points of the calibration model and building structure are represented by 0 and the pixel points of people activity areas are represented by 1. To distinguish the calibration model from the building structure, a new image matrix **I**_match_ is created by Equation (8). The new image matrix **I**_match_ is directly used by the map matching algorithm.

(8) $${\text{I}}_{\text{match}}={\text{I}}_{\text{no}\_\text{model}}-2\times \left({\text{I}}_{\text{model}}⊕{\text{I}}_{\text{no}\_\text{model}}\right)$$

where ⊕ is exclusive-or operation.

**Figure 1.**
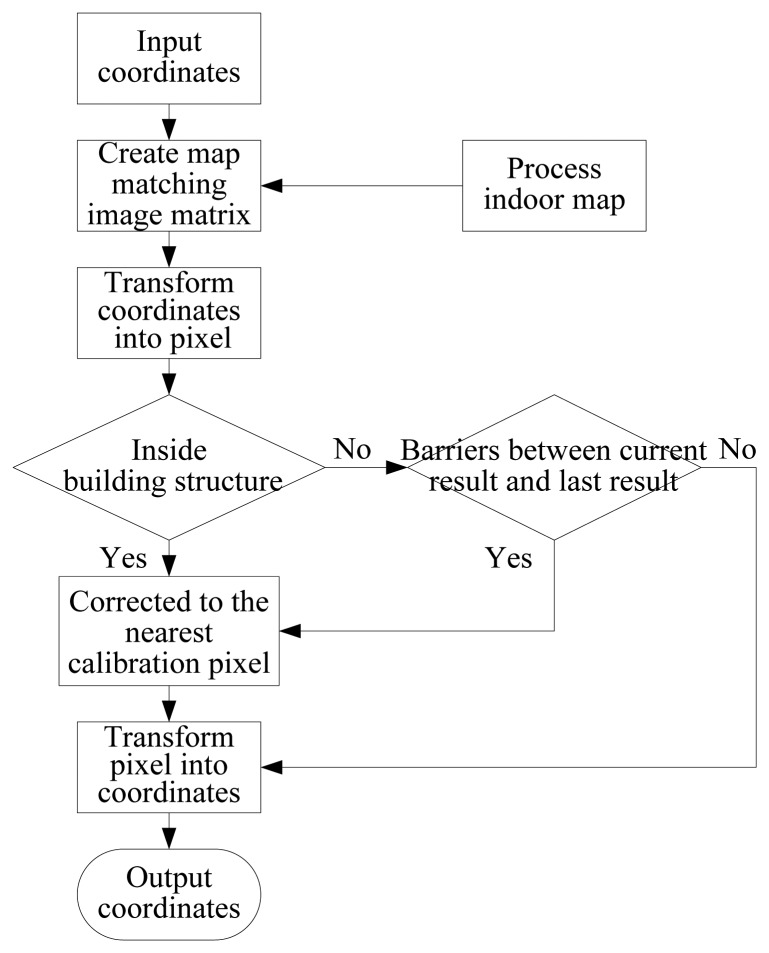
Flow chart of map matching algorithm.

In the new image matrix **I**_match_, the pixel points of people activity areas and building structure are still represented by 1 and by 0, respectively, but the pixel points of calibration model are represented by −1. So the calibration model is recognized by −1 and unreasonable localization results can be corrected to the calibration model. The flow chart of the map matching algorithm is shown in Figure 1 and the algorithm can be generally divided into six steps as follows:

(1) Process the available indoor map information and create accurate building plan image matrix **I**_match_ for map matching.
(2) Transform the *n* th localization coordinates ***U****_n_* = (*x_n_*,*y_n_*) into the corresponding pixel point ${\text{S}}_{n}=\left(p_{n}^{x},p_{n}^{y}\right)$ in the building plan image matrix **I**_match_.
(3) If ${\text{I}}_{\text{match}}\left(p_{n}^{x},p_{n}^{y}\right)=0$, which means that the localization coordinates are located inside the building structure, then go to step 5, otherwise go to step 4.
(4) If building structure barriers exist between the *n* th localization pixel point ***S****_n_* and (*n* − *1*)th localization pixel point ${\text{S}}_{n-1}=\left(p_{n-1}^{x},p_{n-1}^{y}\right)$, then go to step 5, otherwise go to step 6.
(5) Calculate the pixel point ${\text{S}}_{\text{mid}}=\left(\left(p_{n-1}^{x}+p_{n}^{x}\right)/2,\left(p_{n-1}^{y}+p_{n}^{y}\right)/2\right)$ that is the midpoint between ***S****_n_* and ***S****_n_*_−_*_1_*, search the nearest pixel point in the calibration model to the pixel point ***S****_mid_* and then regard the newly searched pixel point as the new localization pixel point ${{\text{S}}_{n}}^{'}=\left(p_{n}^{x'},p_{n}^{y'}\right)$.
(6) Transform the localization pixel point ${{\text{S}}_{n}}^{'}=\left(p_{n}^{x'},p_{n}^{y'}\right)$ into building plan coordinates and output the coordinates ${{\text{U}}^{\prime }}_{n}=\left({x^{\prime }}_{n},{y^{\prime }}_{n}\right)$.

### 3.3. Kalman/Map Filtering for Wi-Fi Fingerprinting Location Sensing

In the area of Wi-Fi fingerprinting location sensing, KF has been applied to process localization results calculated by fingerprinting algorithms. The KF firstly predicts a user's movement state at the present time step by using the previous movement state. When a present measurement result that is a fingerprinting result is obtained, the measurement result is used to correct the predicted movement state. In this process, spatial proximities of consecutive localization results are employed by the KF. However, one drawback of the KF is its linear prediction model cannot precisely predict people movement in indoor environments, so the map matching algorithm is employed by the KMF to nonlinearize the linear prediction process.

The general KF linear process and measurement equations are as follows:

(9) $$\{\begin{array}{ll} {\text{X}}_{n}=\text{A}{\text{X}}_{n-1}+{\text{w}}_{n-1}, & {\text{w}}_{n}\sim N\left(0,\text{Q}\right)\\ {\text{Z}}_{n}=\text{H}{\text{X}}_{n}+{\text{v}}_{n}, & {\text{v}}_{n}\sim N\left(0,\text{R}\right) \end{array}$$

where ***X****_n_* is the state vector, ***A*** is the state transition matrix, ***Z****_n_* is the measurement vector, ***H*** is the measurement design matrix, ***w****_n_* is the process noise, ***v****_n_* is the measurement noise, and ***Q*** and ***R*** are the noise covariance matrices.

The KF operates in two distinct phases: the time update and measurement update. In the time update phase, the new state vector and its uncertainty at the present time step are predicted, which is given by:

(10) $$\left\{ \begin{array}{l} {\hat{\text{X}}}_{\bar{n}}=\text{A}{\hat{\text{X}}}_{n-1}+{\text{w}}_{n-1}\\ {\text{P}}_{\bar{n}}=\text{A}{\text{P}}_{n-1}{\text{A}}^{\text{T}}+\text{Q} \end{array}\right.$$

In the measurement update phase, the predicted vector is corrected and the KF parameters are updated for the next iteration when a measurement result is obtained. The process is denoted by:

(11) $$\left\{ \begin{array}{l} {\text{K}}_{n}={\text{P}}_{\bar{n}}{\text{H}}^{\text{T}}{\left(\text{H}{\text{P}}_{\bar{n}}{\text{H}}^{\text{T}}+\text{R}\right)}^{-1}\\ {\hat{\text{X}}}_{n}={\hat{\text{X}}}_{\bar{n}}+{\text{K}}_{n}\left({\text{Z}}_{n}-\text{H}{\hat{\text{X}}}_{\bar{n}}\right)\\ {\text{P}}_{\bar{n}}=\left(\text{I}-{\text{K}}_{n}\text{H}\right){\text{P}}_{\bar{n}} \end{array}\right.$$

where ***X̂***_n̄_ is the *a priori* estimate of the process, ***X̂***_n_ is the *a posteriori* estimate of the process, ***P***_n̄_ is the covariance matrix of the *a priori* estimate, ***P****_n_* is the covariance matrix of the *a posteriori* estimate, ***K****_n_* is the Kalman gain, and ***I*** is the identity matrix.

The KF linear prediction denoted by Equation (10) causes considerable errors between the predicted locations and actual locations because people move randomly in indoor environments. To predict a user's location more precisely, the proposed map matching algorithm is integrated into the KF prediction process for the nonlinearity of the process. The map matching process for correcting location coordinates is described by a nonlinear function *f*_map_(·) so the nonlinear relationship between the *n* th input coordinates ***U****_n_=* (*x_n_*,*y_n_*)and output coordinates ${{\text{U}}^{\prime }}_{n}=\left({x^{\prime }}_{n},{y^{\prime }}_{n}\right)$ of the map matching function is denoted by:

(12) $${{\text{U}}^{\prime }}_{n}=f_{\text{map}}\left({\text{U}}_{n}\right)$$

Setting the *a priori* estimate of the process ***X̂***_n̄_ as follows:

(13) $${\hat{\text{X}}}_{\bar{n}}={\left[\text{U},\text{V}\right]}_{\bar{n}}^{\text{T}}={\left[x,y,v_{x},v_{y}\right]}_{\bar{n}}^{\text{T}}$$

where ***V***_n̄_ =(*v_x_*,*v_y_*)*_n_*_¯_ is the velocity vector, ***v****_x_* and ***v****_y_* are the velocities in X axis and Y axis, respectively. So after the prediction process is nonlinearized by the map matching algorithm, Equation (13) is changed to:

(14) $${{\hat{\text{X}}}^{\prime }}_{\bar{n}}={\left[{\text{U}}^{\prime },\text{V}\right]}_{\bar{n}}^{\text{T}}={\left[f_{\text{map}}\left(\text{U}\right),\text{V}\right]}_{\bar{n}}^{\text{T}}$$

Subsequently, the formula for calculating the *a posteriori* estimate in Equation (11) is changed to:

(15) $${\hat{\text{X}}}_{n}={{\hat{\text{X}}}^{\prime }}_{\bar{n}}+{\text{K}}_{n}\left({\text{Z}}_{n}-\text{H}{{\hat{\text{X}}}^{\prime }}_{\bar{n}}\right)$$

**Figure 2.**
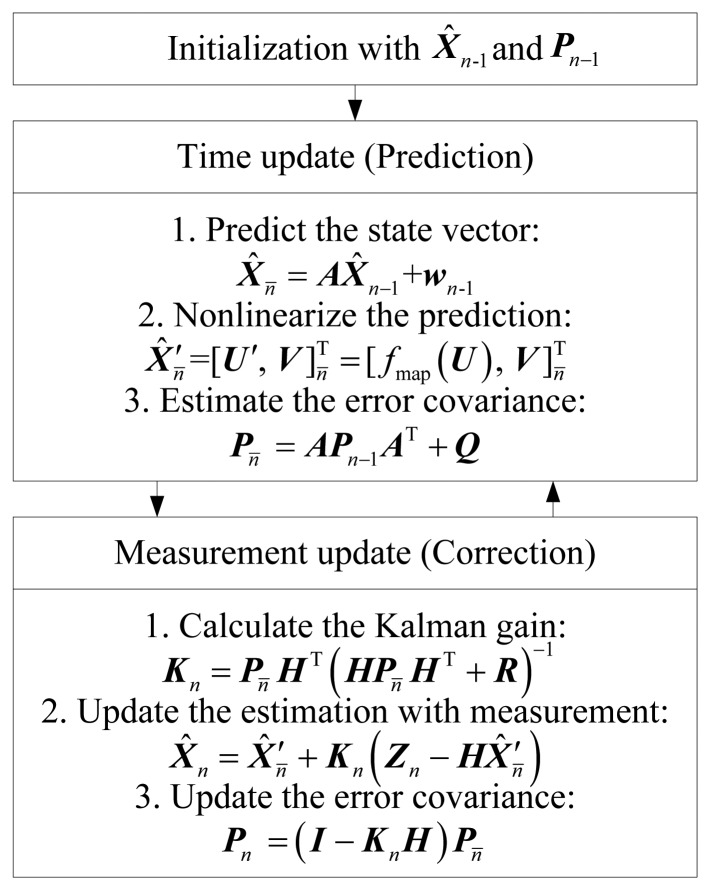
Diagram of Kalman/map filtering.

The diagram of the proposed KMF is shown in Figure 2 as follows.

## 4. Experimental Results and Analyses

### 4.1. Experimental Setup

**Figure 3.**
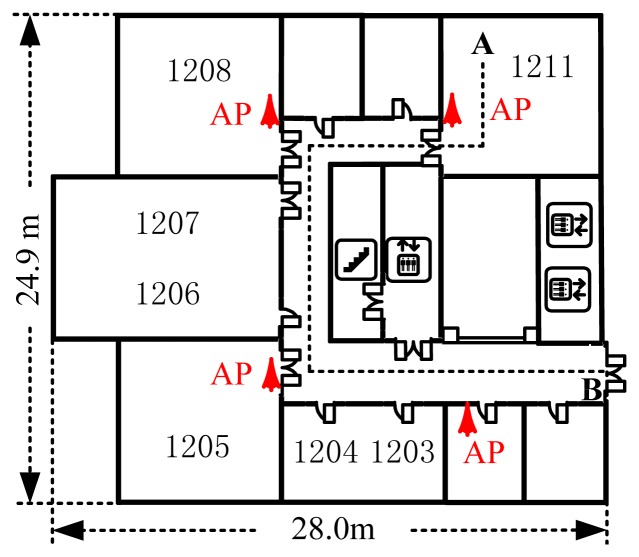
Experimental area plan.

The experimental floor is a rectangular area of 24.9 m × 66.4 m. For communication purposes on the floor, nine Linksys WAP54G APs were deployed. As shown in Figure 3, only four of them were deployed in the 24.9 m × 28.0 m experimental area.

The experimental trajectory spanned Point A in Room 1211 to Point B in the 3 m width corridor. Installed a software named NetStumbler, an ASUS A8F laptop collected RSS samples with a sampling rate of two RSS samples per second. For establishing a radiomap, RSS samples were collected at 91 RPs in Room 1211 and the corridor and 300 RSS samples were recorded within 150 s at each RP. Along the experimental trajectory, a total of 6,500 RSS testing samples were collected at 65 testing points (TPs) with 0.6 m gaps.

### 4.2. Fast Normalized Cross Correlation-Based Fingerprinting Performance

#### 4.2.1. Localization Results of Fast Normalized Cross Correlation-Based Fingerprinting Algorithm

**Figure 4.**
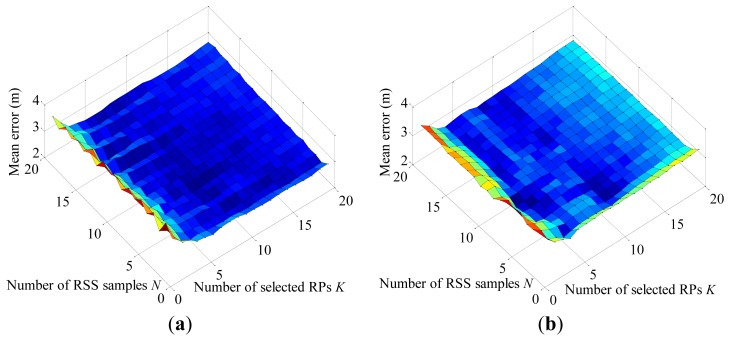
(**a**) Mean error surface of FNCC algorithm with parameters ***N*** and ***K*** varying from 1 to 20, respectively; (**b**) Mean error surface of KNN algorithm with parameters ***N*** and ***K*** varying from 1 to 20, respectively.

The performance of the FNCC algorithm is evaluated with the collected RSS testing data. For comparison, the mean errors of the FNCC and conventional KNN algorithms are calculated with the numbers of on-line RSS samples ***N*** and selected RPs ***K*** varying from 1 to 20, respectively. As shown in Figure 4, the FNCC algorithm outperforms the KNN algorithm. Compared with the KNN algorithm that computes RSS distances between RSS mean samples, the FNCC algorithm makes full use of the available RSS information through exploiting all the on-line RSS samples to avoid useful RSS information loss in RSS mean sample calculations and incorporating RSS variations of RPs in the radiomap denoted by the term $\sqrt{\overset{N}{\underset{j=1}{\text{∑}}}\overset{M}{\underset{i=1}{\text{∑}}}{\left({\text{RSS}}_{i,j,l}-\mu_{l}\right)}^{2}}$ into correlation calculations as weights. As parameter ***K*** increases, the FNCC algorithm performance is more stable than that of the KNN algorithm. When no information is available for setting parameter ***K***, it is easier to set a proper parameter ***K*** for the FNCC algorithm to achieve a comparable accuracy.

**Figure 5.**
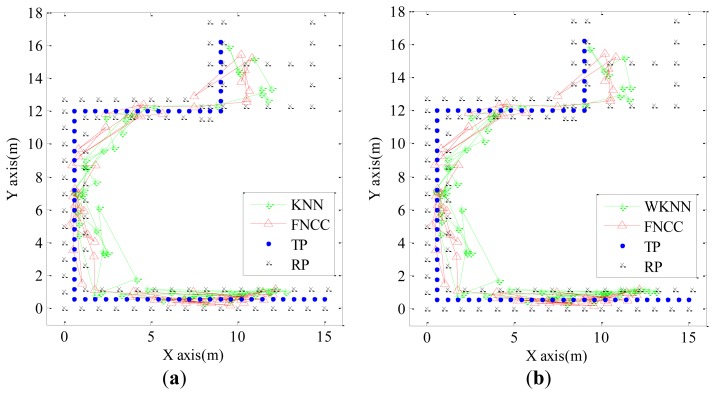
(**a**) Localization results of KNN and FNCC algorithms with parameters ***N*** and ***K*** are set equal to 2 and 7, respectively; (**b**) Localization results of WKNN and FNCC algorithms with parameters ***N*** and ***K*** are set equal to 2 and 7, respectively.

Considering the number and distribution of RPs as well as computational complexity, parameter ***N*** and parameter ***K*** are set equal to 2 and 7, respectively. To simulate the condition that a user moves at a speed of 0.6 m/s along the experimental trajectory, only two RSS testing samples were employed at each TP. The localization results calculated by the KNN, WKNN and FNCC algorithms are shown in Figure 5. The localization results calculated by the FNCC algorithm in Room 1211 and the corridor corners are more accurate than those calculated by the KNN and WKNN algorithms. In this experiment, the mean errors of the KNN, WKNN and FNCC algorithms are 3.00 m, 2.96 m and 2.71 m, respectively. With all these 6,500 RSS testing samples, the mean errors, standard deviations and cumulative probabilities of these fingerprinting algorithms are calculated and listed in Table 2. The FNCC algorithm performance is the same as that of the basic NCC algorithm, which is better than the KNN and WKNN algorithms. The experimental results confirm the advantages of the FNCC algorithm analyzed above and verify the effectiveness of the mathematical operations for simplifying the NCC.

#### 4.2.2. Computational Complexity of Fast Normalized Cross Correlation-Based Fingerprinting Algorithm

Regarding the on-line computational complexity, some terms in both the denominators and numerators of Equations (1), (5) and (6), like (*rss_i,j_* −*μ*_rss_), (*RSS_i,j,l_* − μ_1_) and $\overset{N}{\underset{j=1}{\text{∑}}}\overset{M}{\underset{i=1}{\text{∑}}}{\text{RSS}}_{i,j,l}$, just need to be calculated once for computing one correlation coefficient. Thus, for calculating one localization result, the computational complexities of these algorithms are quantified and listed in Table 3. As mentioned previously, numbers of labeled RPs ***L***, deployed APs ***M***, on-line RSS samples ***N***, and selected RPs ***K*** are equal to 91, 9, 2, and 7, respectively, so the addition/subtraction operation numbers of the KNN, WKNN, NCC with Equation (1), FNCC with Equation (5), and FNCC with Equation (6) are 1,559, 1,571, 11,023, 9,476, and 6,382, respectively. The multiplication/division operation numbers of these algorithms are 821, 835, 5,280, 3,642, and 3,824, respectively, and the numbers of square root operations are 91, 91, 182, 0, and 0, respectively. Compared with NCC with Equation (1), FNCC with Equation (6) saves 4641 addition/subtraction operations, 1456 multiplication/division operations and 182 square root operations. The proposed simplified method effectively reduces the NCC computational complexity. Although addition/subtraction operations and multiplication/division operations of the FNCC with Equation (6) are about three times more than those of the KNN, the KNN needs to calculate 91 square root operations, which is much more complex than the other mathematical operations. Therefore, because of its high accuracy and low computational complexity, the proposed FNCC algorithm is suitable for practical applications.

### 4.3. Kalman/Map Filtering Algorithm Performance for Accuracy Improvement

#### 4.3.1. Map Matching Correction Results

**Figure 6.**
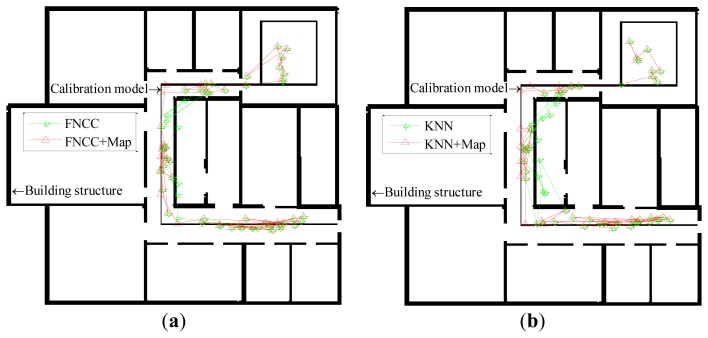
(**a**) Localization results of FNCC algorithm and its corrected results after map matching converted from image matrix. (**b**) Localization results of KNN algorithm and its corrected results after map matching converted from image matrix.

In this paper, the threshold ***T****_threshold_* for converting the gray-scale images into the binary images is set equal to 0.55. The same mobile user experiment shown in Figure 5 is used to verify the effectiveness of the map matching algorithm. The localization results of both the KNN and FNCC algorithms, their corrected results by the map matching algorithm, the building structure, and the calibration model are converted from image matrices and shown in Figure 6 below.

Through map matching, the mean errors of the KNN and FNCC algorithms are reduced from 3.00 m and 2.71 m to 2.96 m and 2.59 m, respectively. With all the 6,500 RSS testing samples, the localization results of the KNN, WKNN and FNCC algorithms corrected by the map matching algorithm are listed in Table 4. The mean errors of the KNN, WKNN and FNCC algorithms are reduced to 2.62 m, 2.57 m and 2.39 m, respectively, and their standard deviations are reduced to 1.90 m, 1.89 m and 1.85 m, respectively. After the map matching corrections, the cumulative probabilities of all these algorithms increase. As shown in Table 2 in Section 4.2.1, the cumulative probability of the FNCC within 2 m and 3 m errors are 45.9% and 68.9%, respectively, which are still better than those of the KNN + Map that are 43.5% and 67.4%, respectively. The FNCC localization results corrected by the map matching algorithm are apparently more accurate than the results of the other algorithms. These experimental results indicate that the proposed map matching algorithm is effective to correct localization results with the indoor map.

#### 4.3.2. Results of Kalman/Map Filtering Algorithm

With the map matching algorithm, the KF linear prediction process is nonlinearized by the proposed KMF. Accurate prediction locations corrected by the map matching algorithm increase the accuracy of the KMF estimates after measurement corrections. Based on the KF theory, the proposed KMF process equation is set as follows:

(16) $${\left[\begin{array}{c} x\\ y\\ v_{x}\\ v_{y} \end{array}\right]}_{n}=\left[\begin{array}{cccc} 1 & 0 & \Delta T & 0\\ 0 & 1 & 0 & \Delta T\\ 0 & 0 & 1 & 0\\ 0 & 0 & 0 & 1 \end{array}\right]\cdot {\left[\begin{array}{c} x\\ y\\ v_{x}\\ v_{y} \end{array}\right]}_{n-1}+\left[\begin{array}{c} \frac{\Delta T^{2}}{2}\\ 0\\ \Delta T\\ 0 \end{array}\begin{array}{c} 0\\ \frac{\Delta T^{2}}{2}\\ 0\\ \Delta T \end{array}\right]\cdot {\left[\begin{array}{c} a_{x}\\ a_{y} \end{array}\right]}_{n-1}$$

where *a_x_* and *a_y_* are the accelerations in X axis and Y axis, respectively. They are set equal to 0.05 m/s^2^. ***w****_n_* ∼ *N*(0,***Q***) is the process noise and $\text{Q}=E\left[{\text{w}}_{n}{\text{w}}_{n}^{\text{T}}\right]$ is computed by the ***w****_n_*.

The KMF measurement equation is given by:

(17) $${\left[\begin{array}{c} z_{x}\\ z_{y} \end{array}\right]}_{n}=\left[\begin{array}{cccc} 1 & 0 & 0 & 0\\ 0 & 1 & 0 & 0 \end{array}\right]\cdot {\left[\begin{array}{c} x\\ y\\ v_{x}\\ v_{y} \end{array}\right]}_{n}+v_{n}$$

where ***z****_x_* and ***z****_y_* are the measurement coordinates in X axis and Y axis, respectively. ***v****_n_* ∼ *N*(0,***R***) is the measurement noise and $\text{R}=E\left[{\text{v}}_{n}{\text{v}}_{n}^{\text{T}}\right]$ is set equal to $\left[\begin{array}{cc} 6 & 0\\ 0 & 6 \end{array}\right]$ according to the fingerprinting results.

**Figure 7.**
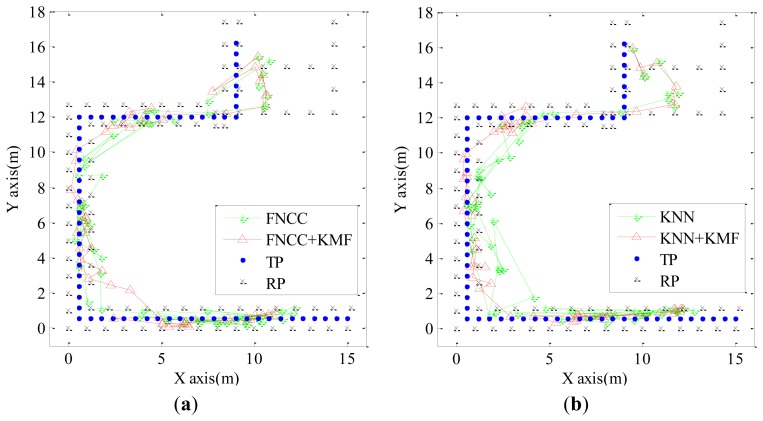
(**a**) Localization results of FNCC and FNCC + KMF; (**b**) Localization results of KNN and KNN + KMF.

Figure 7 shows the experimental results of the KNN and FNCC mentioned in Section 4.2 and their processed results by the KMF. The processed results by the KMF are more consecutive and reasonable than those of the KNN and FNCC because they are corrected with the indoor map and spatial proximities of consecutive localization results. This illustrates that the KMF has the advantages of both the map matching algorithm and KF. Thus, the KMF outperforms each of the two algorithms. In the experiment shown by Figure 7, the mean errors of the FNCC + KMF and KNN + KMF are 1.59 m and 1.73 m, respectively, which is better than the map matching results mentioned in Section 4.3.1 and also better than the mean errors of the results processed by the KF that are 2.55 m and 2.83 m, respectively.

**Figure 8.**
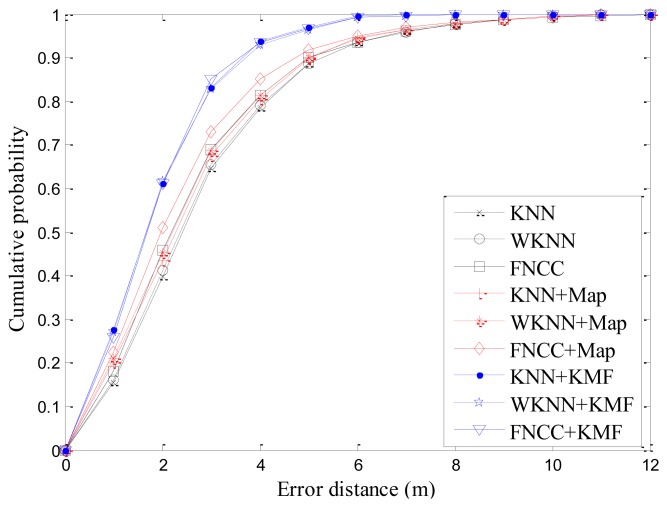
Cumulative probabilities of various algorithms.

With all the 6,500 RSS testing samples, the mean errors of KNN + KF, WKNN + KF and FNCC + KF are 2.65 m, 2.65 m and 2.41 m, respectively. By comparison, the mean errors of the KNN + KMF, WKNN + KMF and FNCC + KMF are 1.88 m, 1.90 m and 1.87 m, respectively. Their standard deviations are 1.24 m, 1.27 m and 1.24 m, respectively. These results indicate that the KMF outperforms the KF. Combined with Table 2 in Section 4.2.1 and Table 4 in Section 4.3.1, the results of the KNN, WKNN, FNCC, and their processed results by the proposed map matching and KMF algorithms are listed in Table 5. With the proposed KMF algorithm, the mean errors of the KNN, WKNN and FNCC decrease 32.1%, 30.7% and 28.1%, respectively. The cumulative probability curves of all these algorithms are also shown in Figure 8. The cumulative probabilities of the localization results processed by the KMF are much higher than those of the other algorithms. Specifically, the cumulative probabilities of the FNCC + KMF within 2 m and 3 m errors reach 61.3% and 85.1%, respectively. These results demonstrate that the proposed KMF is remarkably effective to improve location sensing accuracy by making use of indoor map information and spatial proximities of consecutive localization results.

## 5. Conclusions

In this paper, a KMF-aided FNCC Wi-Fi fingerprinting indoor location sensing system is proposed. Because the NCC is able to make full use of off-line RSS variations of RPs and all the collected on-line RSS samples, the NCC fingerprinting algorithm outperforms the conventional KNN and WKNN fingerprinting algorithms that calculate a localization result with only an on-line RSS mean sample. However, the computational cost of the basic NCC algorithm is very expensive, so an FNCC algorithm is developed. It greatly reduces computational complexity while maintaining the same high accuracy as the basic NCC algorithm. Through integrating a proposed map matching algorithm into the KF, a KMF is proposed to improve location sensing accuracy. Using available indoor map information, the proposed map matching algorithm corrects unreasonable locations computed by the KF linear state prediction process to a calibration model, which represents pedestrian walkways. Based on the corrected locations, the KMF can calculate more accurate localization results using measurement data than the KF. The experimental results confirm both that the FNCC significantly reduces computational complexity of correlation computations without accuracy decreases and that KMF effectively improves location sensing accuracy by using indoor map information and spatial proximities of consecutive localization results.

## Figures and Tables

**Table 1.** Key symbols used in this paper.

**Notations**	**Definition**
*L L*	Number of labeled RPs
*M*	Number of deployed APs
*N*	Number of on-line RSS samples for computing one result
*K*	Number of selected RPs
**rss**	On-line RSS matrix for computing localization coordinates
**RSS***_l_*	Created RSS matrix of *l* th RP
*r_l_*	Correlation coefficient between RSS matrices **rss** and **RSS***_l_*
*rss_i,j_*	RSS numerical value in *i*th row and *j*th column of matrix **rss**
*RSS_i,j,l_*	RSS numerical value in *i*th row and *j*th column of matrix **RSS***_l_*
**I**_match_	Image matrix for map matching

**Table 2.** Performance comparison of KNN, WKNN, NCC, and FNCC algorithms.

**Algorithms**	**Mean Error (m)**	**Standard Deviation (m)**	**Cumulative Probability (%)**	
			**Within 2 m**	**Within 3 m**
KNN	2.77	1.91	39.6	64.7
WKNN	2.74	1.91	41.2	65.4
NCC with Equation (1)	2.60	1.93	45.9	68.9
FNCC with Equation (5)	2.60	1.93	45.9	68.9
FNCC with Equation (6)	2.60	1.93	45.9	68.9

**Table 3.** Computational complexity comparison.

**Algorithms**	**Addition/Subtraction**	**Multiplication/Division**	**Square Root**
KNN	*L*(2*M*−1) + 2*K*−2	*LM*+ 2	*L*
WKNN	*L*(2*M*−1) + 4*K*−24	*LM*+*2K* + 2	*L*
NCC with Equation (1)	*L*(7*M*−5) + 2*K*−2	*LM*(3*MN*+4) +2	*L*
FNCC with Equation (5)	*L*(6*M*−4)+ 2*K*−2	*LM*(2*MN*+4) +2	0
FNCC with Equation (6)	*L*(4*MN*−2) + 2*K*−2	*LM*(2*MN*+6) +2	0

**Table 4.** Comparison of map matching results.

**Algorithms**	**Mean Error (m)**	**Standard Deviation (m)**	**Cumulative Probability (%**)	
			**Within 2 m**	**Within 3 m**
KNN + Map	2.62	1.90	43.5	67.4
WKNN + Map	2.57	1.89	45.4	68.7
FNCC + Map	2.39	1.85	51.0	73.0

**Table 5.** Comparison of KNN, WKNN, FNCC, and their processed results by map matching and KMF algorithms.

**Algorithms**	**Mean Error (m)**	**Standard Deviation (m)**	**Cumulative Probability (%**)	
			**Within 2 m**	**Within 3 m**
KNN	2.77	1.91	39.6	64.7
WKNN	2.74	1.91	41.2	65.4
FNCC	2.60	1.93	45.9	68.9
KNN + Map	2.62	1.90	43.5	67.4
WKNN + Map	2.57	1.89	45.4	68.7
FNCC + Map	2.39	1.85	51.0	73.0
KNN + KMF	1.88	1.24	61.0	83.0
WKNN + KMF	1.90	1.27	61.6	83.0
FNCC + KMF	1.87	1.24	61.3	85.1
